# Retinal Capillary Plexus Pattern and Density from Fovea to Periphery Measured in Healthy Eyes with Swept-Source Optical Coherence Tomography Angiography

**DOI:** 10.1038/s41598-020-58359-y

**Published:** 2020-01-30

**Authors:** Carlo Lavia, Pedro Mecê, Marco Nassisi, Sophie Bonnin, Jennifer Marie-Louise, Aude Couturier, Ali Erginay, Ramin Tadayoni, Alain Gaudric

**Affiliations:** 1 Université de Paris, Ophthalmology Department, AP-HP, Hôpital Lariboisière, F-75010 Paris, France; 2 Institut Langevin, ESPCI Paris, CNRS, PSL University, 1 rue Jussieu, 75005 Paris, France; 3 Sorbonne Université, Institut national de la santé et de la recherche médicale, Centre national de la recherche scientifique, Institut de la Vision, Paris, 75012 France; 4 Centre Hospitalier National d’Ophtalmologie des Quinze-Vingts, DHU Sight Restore, Inserm-Direction Générale de l’Offre de Soins, CIC1423 Paris, France; 5 Pitié Salpêtrière Hospital, Department of Ophthalmology, Sorbonne Université, Paris, 75013 France

**Keywords:** Anatomy, Medical research

## Abstract

Optical coherence tomography angiography is evolving towards wider fields of view. As single widefield acquisitions have a lower resolution, preventing an accurate segmentation of vascular plexuses in the periphery, we examined the retinal vascularisation from the macula to the periphery in all retinal quadrants, using 3 × 3-mm volume scans, to obtain montages with sufficient image resolution up to 11 mm from the foveal centre. Images were qualitatively and quantitatively analysed, using C- and B-scan approaches to calculate the capillary density (CD) and the interplexus distance (IPD). Three vascular plexuses (*i.e*., superficial vascular plexus: SVP, intermediate capillary plexus: ICP, and deep capillary plexus: DCP) were observed up to the mid-periphery in all sectors. The CD of the SVP decreased from about 5 mm of eccentricity, along with ganglion cell density decrease. The CD of the ICP progressively decreased from the fovea towards the periphery, along with the retinal thinning and then vanished from 8 to 9 mm of eccentricity, becoming undetectable beyond. This ICP disappearance resulted in an increased IPD between the SVP and the DCP in an area known to be frequently affected by capillary drop-out in diabetic retinopathy. The DCP only showed a slightly decreased CD towards the retinal periphery.

## Introduction

Optical coherence tomography angiography (OCTA) is becoming an integral part of the multimodal imaging of retinal vascular diseases, as a three-dimensional (3D), repeatable, and non-invasive tool. OCTA can distinguish *in vivo* the macular retinal capillary plexuses, with results similar to those from histological studies^1,2^. In the assessment of macular diseases, OCTA has allowed obtaining satisfying results, comparable or even better than those obtained with dye angiography^3–5^.

The recent availability of wide-field (WF) OCTA has allowed recording retinal capillary maps up to the equatorial region^6^. The potential advantages of this technique when evaluating vascular diseases have recently been shown: non-perfusion areas and preretinal new-vessels are more easily detected due to the high contrast between flow and no-flow areas, the absence of dye leakage and the location of abnormal vessels in or on the retina^7–9^.

Detecting and assessing the surface of capillary non-perfusion areas is a critical prognostic factor in many retinal vascular diseases, especially in diabetic retinopathy and retinal vein occlusion^10–13^. This objective has been partially met using ultra-widefield fluorescein angiography (FA), but its resolution in the periphery is insufficient to accurately measure capillary non-perfusion areas compared to WF-OCTA^14^. OCTA also has the advantage of examining separately each of the different capillary plexuses as well as providing a detailed analysis of the foveal avascular zone (FAZ)^15,16^.

Thus, it becomes important to better know the morphology, distribution and density of the different capillary plexuses from the fovea to the periphery. However, although WF-OCTA provides an image of the whole retinal capillary network that is qualitatively better than FA images, the resolution and segmentation of each capillary plexus with wider scans (i.e., 12 × 12-mm C-scans) is not sufficient to reliably assess the capillary plexuses and measure the capillary density (CD)^17,18^.

The aim of this study was to evaluate the normal retinal vascularisation from the fovea to the far periphery. To overcome some of the limitations of wider scans, montages of small-field (3 × 3-mm) OCTA C-scans were used to provide a better resolution and fewer segmentation artefacts.

## Methods

### Demographics

This study, conducted in the Ophthalmology department of Lariboisière Hospital, Paris University, Paris, France, was approved by the Ethics Committee of the French Society of Ophthalmology (IRB 00008855 Societe Française d’Ophtalmologie IRB#1) and adhered to the tenets of the Declaration of Helsinki. Informed consent was obtained from all subjects.

Subject inclusion criteria were absence of systemic disease, media opacities, history of eye surgery, and retinal diseases or glaucoma and having a refractive error ranging between −3 and +2 dioptres.

### OCTA device

OCTA examination was performed with the 100 kHz Plex® Elite 9000 Swept-Source OCTA device (Carl Zeiss Meditec Inc., USA) equipped with a prototype software (Software version 1.9.0.39060). The centre wavelength ranged between 1,040 and 1,060 nm with a bandwidth of 100 nm, an A-scan depth of 3.0 mm in tissue, an axial optical resolution of about 6.3 μm and a transverse resolution, calculated at the beam size of the pupil, estimated at ≈20 μm.

For this study, 3 × 3-mm OCTA scans were acquired for each subject. Each 3 × 3-mm volume consisted of 300 B-scans of 300 A-scans repeated four times and captured with FastTrac eye motion correction software (Carl Zeiss Meditec, Inc, USA).

### OCTA acquisition

Each 3 × 3-mm volume was acquired from the foveal centre to the retinal periphery to create a horizontal and a vertical band passing through the fovea. Briefly, after the central fovea acquisition, the scanning cursor was moved in the desired direction using the live fundus image as a reference and scans were acquired to ensure a sufficient overlay between adjacent volumes (i.e., about 40%).

The horizontal band passed through the ONH.

### Image montage

We used a 12 × 12-mm volume acquisition as a reference to obtain a wide-field image. Then, consecutive 3 × 3-mm volumes were manually superimposed starting from the foveal centre towards the retinal periphery in the four directions. Correct image overlaying was verified by two experienced examiners (C.L. and A.G.) based on the en face pattern of the superficial vascular plexus shown by the 12 × 12-mm volume.

**Figure 1:**
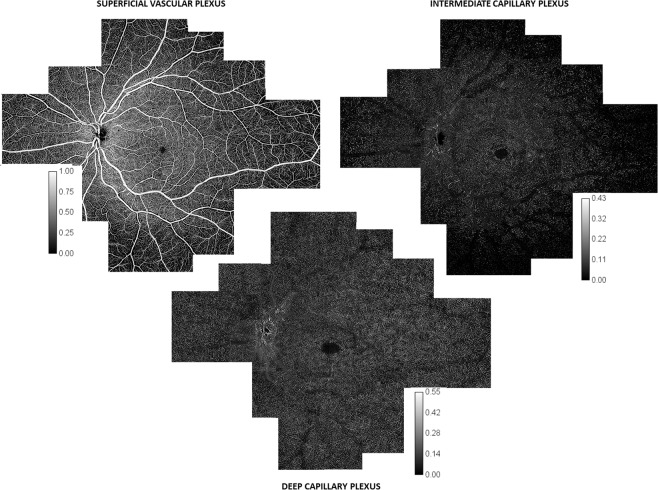
C-scan wide-field montages of the three capillary plexuses. 3 × 3-mm C-scan montage of the superficial vascular plexus (SVP, top left), intermediate capillary plexus (ICP, top right) and deep capillary plexus (DCP, bottom), obtained from 63 C-scans. The SVC was composed of large vessels going from and to the optic nerve head (ONH), the radial peripapillary capillary plexus (RPCP) and the superficial vascular plexus (SVP). The RPCP was detectable as a circle around the ONH and then along the superior and inferior temporal vascular arcades. Inside the vascular arcades, the SVP capillary density (CD) appeared relatively constant, except for the foveal avascular zone (FAZ). Moving towards the periphery, the whole SVP was darker, due to the absence of the RPCP and the decrease in CD. The ICP had an almost constant CD inside the vascular arcades. Outside the arcades, its CD progressively decreased, and then rapidly became almost undetectable at similar distances from the foveal centre in the superior, inferior and temporal sectors and more peripherally in the nasal sector. The DCP was seen as a network of capillaries with an anastomotic lobular capillary organisation, centred by post-capillary venules. From the foveal centre to the retinal periphery, both its CD and pattern appeared relatively constant. The intensity level was normalised and is represented by a calibration bar for each image.

For each subject, 25 3 × 3-mm scans were acquired on average. In two subjects, other volumes were acquired outside the horizontal and vertical bands in order to cover a higher surface, using 63 3 × 3-mm volumes on average (Fig. 1).

The analysis of each band was performed according to the Polyak’s anatomical definition of the various zones of the retina from the fovea to the far periphery^19^. Briefly, the radius of the foveola is 350 µm, and that of the foveal floor is 400 µm. The parafovea is a ring ranging between 400 and 1250 µm from the centre, the perifovea between 1250 and 2750 µm, the near periphery between 2750 and 4250 µm, the mid periphery between 4250 and 7250 µm and the far periphery beyond 7250 µm from the centre.

En face OCTA images were generated by the complex optical microangiography (OMAGc) algorithm which uses the changes in both intensity and phase information between the sequential B-scans at the same location to detect a flow signal^20^.

The device provides automatic segmentation at the ILM and retinal pigment epithelium (RPE) and then derives the relative positions of the IPL and OPL. After image acquisition, the correctness of retinal layer segmentation was verified, by scrolling the cursor through all the B-scans. A manual correction was performed in case of segmentation errors and if they were not greater than 5% of the total volume (i.e., 15 B-scans). In case of excessive segmentation errors or image tilting impeding a correct delineation of retinal layers, the OCTA scans were rejected, and the acquisition was repeated. Only scans showing a signal strength ≥9/10 were considered for the analysis to ensure a proper and homogeneous image quality. In the case of a signal strength <9/10, the scans were repeated. On average, two scans were rejected for each subject for quality reasons.

### Retinal segmentation

#### OCTA C-scan

The device automatically provided an image of the SVP and DVP, using default slabs, the inner and outer boundaries of which were set at the ILM and IPL-INL (SVP) interface and the IPL-INL and OPL-ONL (DVP) interfaces. In this study, custom slabs were used to visualise the three vascular plexuses (SVP, ICP and DCP), according to histological and OCTA studies. In each OCTA volume, the C-scans were selected based on the location of the flow signal on the B-scans and their en face appearance and to provide the best identification of each vascular plexus.

In particular, the SVP was detected between the boundaries set at the ILM and inner two-thirds of the IPL, the ICP between the outer boundary of the SVP and a boundary just behind the INL-OPL interface, and the DCP between the outer boundary of the ICP and a boundary just behind the OPL-ONL interface. There was no overlap between slabs. As the thickness of the retina decreased towards the periphery, the thickness of each slab used to display each capillary plexus also varied accordingly (Supplementary Data 1).

### Calculation of capillary density and interplexus distance

Images were analysed to obtain CD from the OCTA scans. Capillary density was calculated using both C-scan and B-scan computation methods. In the SVP we measured the CD after excluding the large retinal vessels. However, capillaries of the RPCP near the optic disc on the horizontal bands and at the temporal vascular arcades on the vertical bands were included in the analysis.

### C-scan procedure

**Figure 2:**
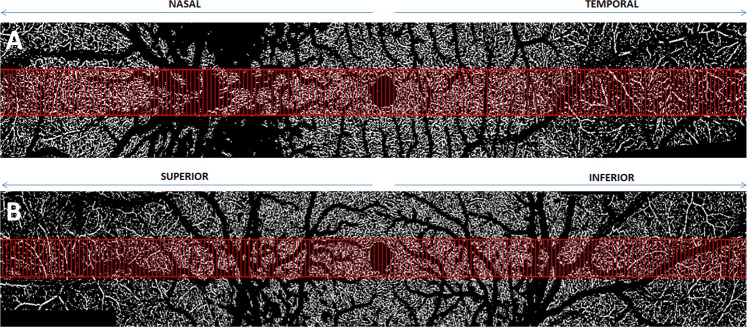
Capillary density analysis on C-scan montages. Example of one horizontal (**A**, nasal to temporal) and one vertical (**B**, superior to inferior) OCTA C-scan montages from the superficial vascular plexus (SVP) used to calculate the capillary density (CD). Large vessels going from and to the optic nerve head at the SVP were masked by imaging processing for all images generated by the OCTA C-scans, as described in the methods section. The same mask was applied for the intermediate capillary plexus (ICP) and deep capillary plexus (DCP) OCTA C-scans. Red rectangles represent the area (0.1 × 0.8 mm²) where the CD was calculated.

**Figure 3:**
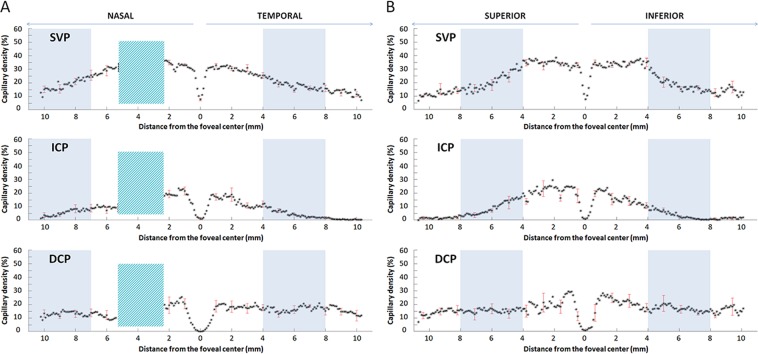
C-scan analysis of the capillary density in the three plexuses. Mean and standard deviation (SD) of the capillary density (CD, %, y-axis) of the ten eyes analysed in the superficial vascular plexus (SVP, top), intermediate capillary plexus (ICP, middle) and deep capillary plexus (DCP, bottom). Points are reciprocally spaced 0.1 mm apart; the SD is given every 10 points. CD values were calculated on the C-scans at different eccentricities from the foveal centre (0 mm, x-axis) along a horizontal band (**A**, nasal to temporal) and a vertical band (**B**, superior to inferior). Values on the x-axis indicate distances in mm from the foveal centre, arrows on the top (nasal-temporal and superior-inferior) indicate the explored sector. On the horizontal band, a  green box, located nasally to the foveal centre, represents the optic nerve head, which was excluded from the analysis. Within the foveal centre, a small and steep depression was observed in both bands and was more pronounced in the SVP. The blue boxes represent the area where the SVP density starts to decrease.

For each subject, the CD of the SVP, ICP and DCP were measured on the horizontal and vertical bands of montages of 3 × 3-mm C-scans. The following procedure was used as previously described by Campbell^21^. First, areas occupied by larger vessels were excluded from the CD analysis by applying a mask that used an intensity-based thresholding algorithm applied to the SVP C-scan (Gaussian window of 15 × 15 pixels, intensity threshold of 0.4). Then, flow pixels were counted for each C-scan in a 0.1 × 0.8-mm (x × y) sample area and divided by the total number of pixels to obtain the CD. Sample areas that were covered by the larger vessel masks (>40% of the area) were excluded from the analysis (Fig. 2). CD values are reported in Table 1 and in Fig. 3. The descriptive analysis considered the maximum CD values as the highest mean CD values among the 0.1 × 0.8-mm samples analysed.

**Table 1:** Capillary density (%) in each retinal capillary plexus according to the distance from the foveal centre in the four retinal sectors.

	1 mm	2 mm	4 mm	8 mm	10 mm
**Temporal**					
SVP (%)	33.0 ± 1.3	32.7 ± 1.5	25.5 ± 2.1	15.9 ± 2.4	11.7 ± 1.8
ICP (%)	17.2 ± 2.4	19.0 ± 5.0	12.0 ± 2.2	1.9 ± 0.9	0.4 ± 0.1
DCP (%)	18.7 ± 4.4	18.4 ± 4.1	17.7 ± 2.2	15.2 ± 2.9	11.8 ± 3.9
**Nasal**					
SVP (%)	33.2 ± 0.8	35.0 ± 1.8	NA	21.0 ± 2.3	15.2 ± 2.4
ICP (%)	21.5 ± 2.9	19.3 ± 2.8	NA	8.5 ± 3.0	3.9 ± 1.8
DCP (%)	20.5 ± 3.6	19.5 ± 6.0	NA	13.2 ± 2.6	10.3 ± 1.7
**Superior**					
SVP (%)	29.3 ± 2.0	35.8 ± 1.7	36.5 ± 3.5	14.3 ± 2.2	11.5 ± 1.4
ICP (%)	24.8 ± 5.0	24.0 ± 4.3	17.9 ± 3.5	3.3 ± 1.4	1.6 ± 0.9
DCP (%)	29.1 ± 4.0	18.4 ± 7.1	16.7 ± 5.7	16.4 ± 3.3	10.3 ± 2.5
**Inferior**					
SVP (%)	32.5 ± 1.6	34.2 ± 2.2	35.7 ± 2.7	13.6 ± 3.0	10.7 ± 2.6
ICP (%)	22.2 ± 4.4	18.5 ± 4.6	13.9 ± 2.6	0.8 ± 0.5	0.9 ± 0.4
DCP (%)	26.5 ± 3.0	22.9 ± 3.3	16.3 ± 3.5	16.7 ± 2.9	14.8 ± 4.7

SVP: superficial vascular plexus. ICP: intermediate capillary plexus. DCP: deep capillary plexus. Values are expressed as a mean ± standard deviation. mm: millimeter.

### B-scan procedure

The capillary flow peaks were determined by extracting data from the flow overlay on the B-scans. Forty consecutive horizontal B-scans were extracted from each consecutive C-scan in order to perform a continuous analysis from the foveal area to the temporal periphery in all subjects. A correct overlay of the B-scans was achieved based on vessel morphology in the SVP on the C-scans. To identify the depth of each retinal vascular plexus, OCTA volumes were aligned according to the RPE layer. To improve the axial resolution, superficial vessel projection artefacts in the deeper layers were removed using the projection-resolved (PR) OCTA algorithm introduced by Campbell *et al*. and Zhang *et al*.^21,22^. Before measuring the capillary flow peaks, larger vessel (arteries and veins) masks were created to exclude them from the analysis. Larger vessel masks were deducted by filtering the signal using an intensity-based thresholding algorithm directly on each B-scan, using a 20 × 20-pixel Gaussian window. Then, flow pixels were counted for each depth (z) plane of 6 µm in a 0.3 × 0.3-mm² (x × y) sample area and divided by the total number of pixels to obtain the CD, where the unit was the percentage of flow per volume.

**Figure 4:**
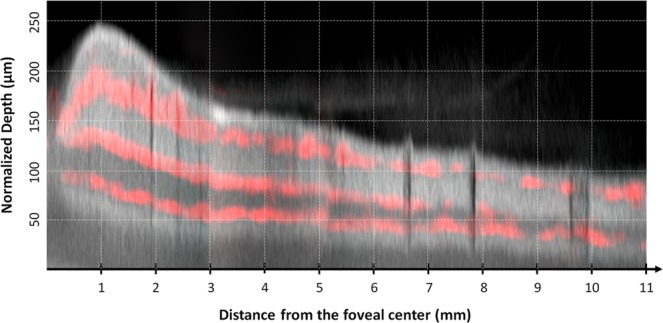
Structural B-scan montage with flow overlay in the temporal sector. Composite image showing the mean flow signal (red) of the superficial vascular plexus (SVP), intermediate capillary plexus (ICP) and deep capillary plexus (DCP) superimposed to an averaged structural B-scan image going from the foveal centre (left) to the retinal periphery (right). A grid composed of dotted lines spaced 50 µm apart on the y-axis and 1 mm apart on the x-axis was added. The structural B-scan showed the retinal thickness profile, from the foveal depression to a rapid thickening in the parafoveal area then followed by a progressive thinning towards the peripheral retina. The upper red line corresponds to the SVP, which was continuous up to the retinal periphery and located within the ganglion cell layer and the superficial part of the inner plexiform layer (IPL). The middle red line corresponds to the ICP, located within the deeper part of the IPL and the more superficial part of the inner nuclear layer (INL). The lower red line corresponds to the DCP, located within the deeper part of the INL and the superficial part of the outer plexiform layer (OPL). Moving towards the periphery, the three vascular plexuses became closer to each other until the ICP vanished. Beyond the limit of the ICP, the interplexus distance, which was of about 24–36 µm between each of the 3 plexuses, increased to 55 µm between the two remaining plexuses, i.e. the SVP and the DCP.

**Figure 5:**
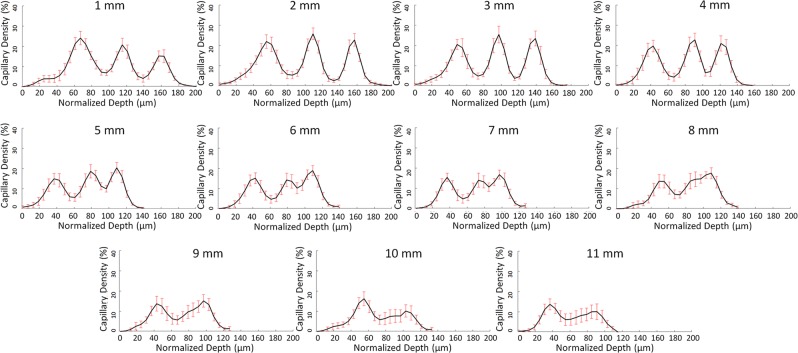
B-scan analysis of the capillary density from the fovea towards the temporal periphery. Mean and standard deviation of the capillary density (CD, %, y-axis) of the ten eyes analysed according to the retinal depth (µm, x-axis) at increasing eccentricities (as indicated in the upper part of each graph) on a horizontal band going from the foveal centre to the temporal periphery. On the x-axis, zero (“0”) corresponds to the retinal surface (i.e., the inner limiting membrane). Capillary density values were calculated on the B-scans. In the first, second, and third lines, data between 1 and 4 mm, 5 and 8 mm, and 9 and 11 mm of eccentricity from the foveal centre are respectively presented. At 1 mm, the highest peak corresponds to the SVP, with a maximum CD located at 67-µm depth from the retinal surface, within the ganglion cell layer. The SVP-ICP and the ICP-DCP distances were 48 µm and 42 µm respectively. At 2, 3 and 4 mm, a progressive increase in the second (intermediate capillary plexus, ICP, with maximum CD located between 109 and 91 µm) and the third capillary plexus (deep capillary plexus, DCP, with maximum CD located between 157 and 121 µm) was observed and accompanied by a flat decrease in CD of the SVP. At 5 mm, a stronger decrease in CD of the SVP was observed, as shown in the C-scan analysis. The decrease could be due to the decrease in ganglion cell density that has been histologically described at this degree of eccentricity. At 7 mm from the foveal centre the distance between SVP and ICP peaks decreased to 36 µm. Between 6 and 7 mm, the peak density of the ICP and the DCP became progressively closer to each other, spaced by 30 µm at 6 mm and by 24 µm at 7 mm, reducing to 18 µm at 8 mm where the two poorly distinguishable plexuses became a flattened peak. From 9 to 11 mm, only two peaks, corresponding to the SVP and the DCP, were detected at a reciprocal distance greater than 50 µm.

**Figure 6:**
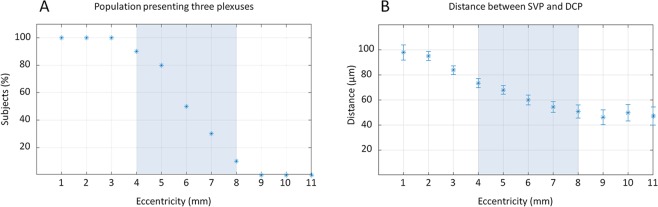
Proportion of subjects presenting three capillary plexuses and interplexus distance. Graphics obtained from the B-scan analysis of the horizontal band of the ten eyes analysed, at increasing eccentricities temporally from the foveal centre. (**A**) Proportion of subjects (%, y-axis) with three distinguishable plexuses from 1 to 11 mm of eccentricity (x-axis). Up to 3 mm, 100% of subjects showed three vascular plexuses. From 4 to 8 mm, the proportion decreased from 80% to 10%, and all subjects showed only two distinguishable plexuses at 9 mm of eccentricity. The blue box, set from 4 to 8 mm, represents the “transitional zone”, where the proportion of subjects with three plexuses progressively decreased. (**B**) Interplexus distance (IPD; µm, y-axis) between the superficial vascular plexus (SVP) and the deep capillary plexus (DCP) between 1 and 11 mm of eccentricity (x-axis). The distance was of about 100 µm at 1 mm. The IPD then progressively decreased to 70 µm at 5 mm and to about 50 µm between 8 and 11 mm. Interestingly, from 9 mm of eccentricity, where the ICP was undetectable in the superior, inferior and temporal sectors, the IPD between SVP and DCP was greater than 50 µm, and slightly decreased up to 10–11 mm and therefore corresponded to the widest area showing higher IPD values compared to more central areas.

Using these data, the vascular plexus location (shown in Figs. 4 and 5) was deducted using a peak identification algorithm (Peakfinder from MatLab)^23^. The parameters used to identify the location of each retinal capillary plexus were: 1) the minimal distance of 9 µm between capillary plexuses, meaning that if two plexuses were closer than 9 µm to each other, they were considered a unique entity, 2) the minimal CD of 0.05% (meaning that areas where the CD was smaller than 0.05 were not considered vascular plexuses). Finally, using the same algorithm, the distance between the SVP and the DCP (shown in Fig. 6B) was computed. Data from Figs. 5 and 6 (B-scan CD, flow peaks count and SVP-DCP distance) are presented as a mean ± standard deviation. The distances between the SVP and the ICP and between the ICP and the DCP, referred to as IPD, were also calculated.

## Results

A total of 10 eyes of 10 healthy subjects (4 men and 6 women, mean age: 31.8 ± 3.4 years [range 25 to 37], mean refractive error: −0.4 ± 1.4 dioptres [range: −3 to +2]) were included.

### Analysis of capillary density based on c-scans

CD values for each plexus are presented in Table 1 and, graphically, in Fig. 3.

**Figure 7:**
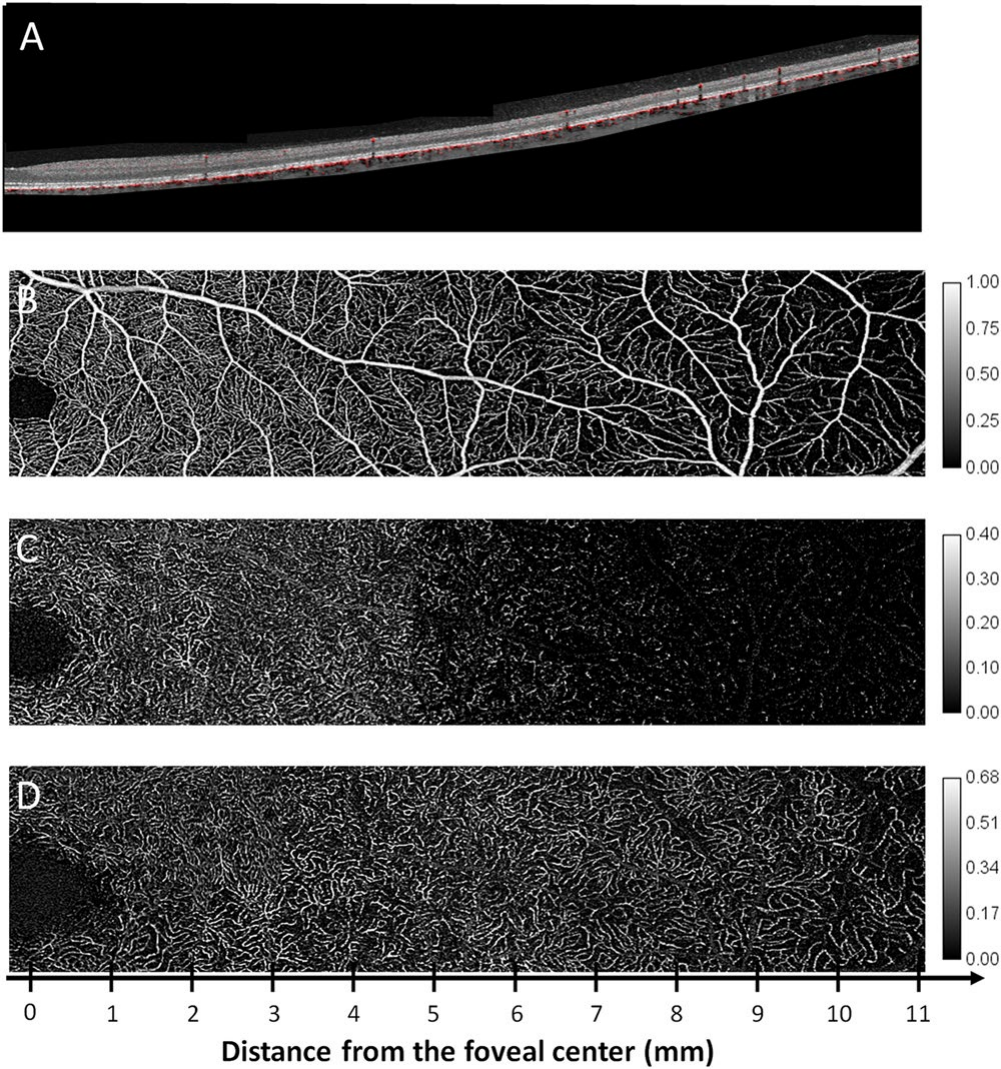
B-scan montage with flow overlay and C-scan montages of the three capillary plexuses. Composite montage of a structural B-scan with flow overlay, after correction of projection artefacts (**A**) and OCTA C-scans from the superficial vascular plexus (SVP), (**B**), intermediate capillary plexus (ICP, **C**) and deep capillary plexus (DCP, **D**). The analysed area goes from the foveal centre to 11 mm in the temporal periphery. The SVP appeared rich in pre-capillary arterioles, post-capillary venules and capillaries up to the borders of the perifoveal area, where they became scarcer, as fewer capillaries were present to perfuse a thinner ganglion cell layer. The ICP was visible up to the border between the perifoveal and mid-peripheral areas, where it rarefied and then disappeared, leaving an almost avascular dark slab in the far periphery. The DCP showed its typical aspect, with a lobular organisation and a planar appearance. It should be noted that up to the far periphery, where it rarefied, it maintained a similar density as that observed in areas close to the foveal centre. The image intensity level was normalised and is shown by the calibration bars.

The analysis was performed on a 22-mm horizontal band from the nasal to the temporal sector passing through the fovea and the lower part of the optic nerve head (ONH) and on a 22-mm vertical band from the superior to the inferior sector passing through the fovea. The appearance of each plexus on the horizontal band from the foveal centre to the temporal retinal periphery is presented in Fig. 7.

### Superficial vascular plexus

The Superficial Vascular Complex (SVC) includes the superficial vascular plexus (SVP) and the radial peripapillary capillary plexus (RPCP). In the SVP, only the CD was considered, after exclusion of the large retinal vessels. On the horizontal band, the highest CD was at about 1.5 mm from the foveal centre on the temporal side and at 2 mm from the foveal centre on the nasal side, i.e., just outside the parafoveal area (34.0 ± 1.8% and 35.0 ± 1.8%, respectively). The CD of the SVP significantly decreased from 2 to 8 mm from the foveal centre, with a loss of 51% on the temporal side and continued to slightly decrease towards the periphery.

On the vertical band passing through the foveal centre, the highest CD values were within the parafoveal border (i.e., at 1,25 mm from the foveal centre) on both sides but values remained almost constant between 0.4 and 4.8 mm superiorly and 4.2 mm inferiorly to the fovea, due to the presence of the RPCP up to the vascular arcades in addition to the SVP.

On the horizontal band, two additional high values were found around the optic nerve head (ONH), on both its nasal and temporal sides, corresponding to the dense RPCP surrounding the ONH.

### Intermediate capillary plexus

The CD of the intermediate capillary plexus (ICP) strongly decreased from 2 mm from the foveal centre, with a loss of about 75% from the parafovea to the mid-periphery, reaching CD values of less than 5% at 5.6 mm temporally, 8.9 mm nasally, 6.5 mm superiorly and 5.5 mm inferiorly to the foveal centre.

Then, the ICP CD progressively vanished, with values of less than 2% in all directions, from 7.9 mm temporally, 10 mm nasally, 8.5 mm superiorly and 7 mm inferiorly to the fovea. Between 2 and 8 mm from the foveal centre, on the temporal side, the mean decrease in ICP CD was 90%. The limits of the ICP had an oval shape with a horizontal major axis centred on the fovea on the *en-face* montage (Fig. 1).

### Deep capillary plexus

The deep capillary plexus (DCP) showed a planar, lobular pattern with units centred by small vortex venules, which was relatively stable from the fovea to the retinal periphery within both the horizontal and vertical bands. Unlike other plexuses, CD values remained stable around 20% from the perifovea to the near periphery. More peripherally, CD slowly decreased to 12% up to 10 mm from the foveal centre. A mean decrease in CD by only 17% was observed between 2 and 8 mm from the foveal centre on the temporal side.

### Analysis of the number of capillary plexuses and interplexus distance based on b-scans

The B-scan analysis was performed on the temporal scans of all subjects, between 1 and 11 mm from the foveal centre. The capillary distribution according to the retinal depth at increasing distances from the foveal centre is presented in Fig. 4 and, graphically, in Fig. 5 (mean and standard deviation of the studied population). Each capillary plexus appeared on our measurements as a flow peak. The depth of each capillary plexus was calculated from the inner limiting membrane (ILM). At 1 mm from the foveal centre, three flow peaks were observed, respectively representing the location of the SVP, the ICP and the DCP. The highest flow values of the SVP, the ICP and the DCP were found at 1, 2 and 3 mm from the foveal centre, respectively. Three distinct flow peaks were detectable between 1 and 7 mm from the foveal centre, while only two, the SVP and the DCP, were detectable at about 8 mm from the foveal centre (Fig. 6A).

We also measured the interplexus distance (IPD), corresponding to the distance between the peaks of the capillary plexuses on the B-scans (Fig. 5). At 1 mm temporal to the foveal centre, the SVP-ICP and the ICP-DCP distances were 48 µm and 42 µm, and they slightly decreased to about 36 µm (SVP-ICP) and to 24 µm (ICP-DCP) in the mid-periphery, up to 7 mm from the foveal centre. This was not the case further in the periphery between 8 and 10 mm from the foveal centre, where a progressive retinal thickness reduction was observed, together with the reduction from 3 to 2 flow peaks (SVP-DCP) which were spaced up to 55 µm, i.e. greater than the distance between each of the 3 capillary plexuses from the foveal centre to the mid-periphery (Fig. 5). The distances between the SVP and the DCP according to the eccentricity from the foveal centre are presented in Fig. 6B.

## Discussion

In this study, we took advantage of the high resolution provided by SS-OCTA at 100 KHz to explore the peripheral retinal circulation. Widefield SS-OCTA allows visualising the retinal vasculature in 3D, even far from the macular area. However, single widefield acquisitions have the disadvantage of a lower transverse resolution compared to narrower field acquisitions^7,17^. Moreover, it is often challenging to obtain a correct segmentation of each vascular plexus on these acquisitions when exploring the retinal periphery.

Therefore, instead of using the wide scans provided by the device to assess the retinal periphery, we used its capacities to explore the retina up to the equator but with 3 × 3-mm (about 10° × 10°) montages to map the retinal capillary plexuses in order to keep the best possible resolution and to reduce the risk of low OCT signal artefacts as recently described^17^. In this way, we obtained 3 × 3-mm C-scans in which the B-scans were spaced 10 µm apart, compared to 24 µm apart in the 12 × 12-mm C-scans. Compared to a 12 × 12-mm acquisition volume, on a 3 × 3-mm OCTA scan the resolution of the retinal capillaries was better, their course was followed in all the three plexuses, and it was easier to recognize the peculiar pattern of each plexus from the fovea to the equator in all directions. The difference was especially marked for the intermediate and deep capillary plexuses (Supplementary Data 2). It was then possible to document the progressive attenuation of the ICP as well as the fourth level of the RPCP around the optic disc^4^ (Fig. 1). We also studied the retinal flow signal on both the C- and B-scans to confirm our findings with greater confidence (Figs. 3, 5).

Our main finding was that the ICP progressively decreased in density towards the periphery and then even vanished, resulting in only two plexuses, the SVP and the DCP. The ICP was no longer detectable at about 8–9 mm from the fovea in the superior, temporal, and inferior quadrants, and at about 9–10 mm from the fovea in the nasal quadrant. The 2 other plexuses were subjected to fewer changes in the SVP, and even less in the DCP (Fig. 1).

The progressive attenuation and disappearance of the ICP have also been observed by Campbell *et al*. on the horizontal axis in the temporal part of the fundus^21^. On the contrary, Hirano *et al*. have not observed the disappearance of the ICP in the periphery based on the flow analysis on OCTA B-scans but their measurements were limited at 22.5° of eccentricity (about 7.5 mm in an emmetropic eye), while in our study, the ICP started to vanish beyond 8–9 mm of eccentricity^24^ (Figs. 4 and 7).

The progressive disappearance of the ICP beyond the mid-periphery could be due to a decrease in ganglion cell layer (GCL) and inner nuclear layer (INL) thickness as well as in their plexiform layers towards the periphery. We could assume that the ICP is needed to supply the inner plexiform layer (IPL) and the inner part of the INL, where the SVP alone cannot provide enough oxygen to both the thick and dense GCL and the IPL. However, the ICP is no longer needed in areas where the GC are not only reduced to a monolayer but become sparser, as seen in the retinal periphery, along with the decreased thickness of the INL and IPL. Indeed, under such conditions, the SVP would be again able to supply the IPL and the inner part of the INL.

Studies on GC density topography in the fundus have shown a rapid decrease in GC density between 1 and 3 mm from the foveal centre and then a progressive decrease from about 7,000 cells/mm^2^ at 3 mm to 800 cells/mm^2^ at 8 mm and 500 cells/mm^2^ at 10 mm on the temporal side, while GC were 3 times more numerous in the nasal periphery than in corresponding areas of the temporal periphery^19,25,26^. Beyond 8 to 10 mm, the GCL becomes discontinuous, the cells are few in number and separated by long gaps^27^. It was in this area that the ICP progressively vanished in our study. Our last observation concerning the ICP was that its limits had an oval shape with a longer horizontal axis, very similar to the GC distribution^25,26^.

The progressive vanishing of the ICP in the periphery should not be understood as a merge with the DCP^21^. It was rather related to a decrease in its IPD with the DCP that was associated with the reduction in retinal depth towards the periphery, making the presence of two capillary plexuses unnecessary in the deeper part of the inner retina (Figs. 4, 6B).

The two other capillary plexuses (the SVP and the DCP) remained present in the whole field examined, i.e. up to the equator, but the SVP density decreased progressively, while the DCP density was more stable with only a slight decrease beyond 10 mm of eccentricity. The CD of the SVP decreased by about 30–35% between 2 and 5 mm from the foveal centre and continued to slightly decrease at 8 mm from the foveal centre. The CD of the DCP decreased only moderately towards the periphery, with a reduction by less than 20% between 3 and 8 mm from the fovea. Interestingly, the outer nuclear layer thickness and the photoreceptor density (cones and rods) have a similar behaviour^27,28^. The INL thickness and the bipolar cell density also decrease in the same proportion as for the photoreceptors^26,27^. The DCP is supposed to supply oxygen to the outer plexiform layer (OPL) and the outer part of the INL but its role in photoreceptor oxygenation is also debated, both under physiological and pathological conditions. Linsenmeier and Zhang, using oxygen-sensitive microelectrodes and oximetry, have found that under both dark and light conditions, the choroidal and retinal circulation could supply, in different proportions, the metabolic demand of photoreceptors^29^. Nesper *et al*., in a cohort of patients with diabetic retinopathy, have observed using adaptive optics and OCTA photoreceptor changes in areas of capillary dropout in the DCP, suggesting that the integrity of the DCP is needed for photoreceptor metabolism^30^. Assuming that retinal capillaries are present in areas where there is a cellular demand, the small decrease in photoreceptor and INL density towards the periphery could explain the relative stability of the DCP density in this area^26^.

The consequences of the progressive rarefaction of the ICP in the mid-periphery in various retinal vascular diseases are unknown. However, it should be noted that, in diabetic retinopathy, the capillary dropout starts preferentially in this transitional zone, a preferred location that has never been explained until now, but where the ICP vanishes^31–33^. It is noteworthy that beyond the limit of the ICP, the IPD, which was initially of about 24–36 µm between each of the 3 plexuses, increased to 55 µm between the SVP and the DCP while the density of the SVP continued to decrease, and this could have resulted in some vulnerability in the neurovascular coupling in this area.

This study has some limitations. The sample size, composed of young healthy subjects with no ocular disease and no refractive disease, is small and further studies in larger cohorts of healthy and diseased subjects are therefore needed to confirm these findings. The acquisition of consecutive 3 × 3-mm OCTA images was standardised but not automated, and this could be a possible source of error in measuring the distances from the foveal centre. However, we paid attention to respect the vascular anatomy based on fundus and C-scan images, and these montages provided a better resolution of the capillary network than 12 × 12-mm acquisitions. The 3 × 3-mm montages cannot be commonly used but they have provided useful information for the next generation of faster SS-OCTA, which will provide more details on capillaries on wider C-scans. The segmentation boundaries used to show the capillary plexuses were based on C-scans and adjusted to provide a more accurate view of each capillary plexus. This strategy could have introduced potential errors in modifying CD values. However, the flow was also analysed on the B-scans and the results supported our C-scan findings. The distance between each capillary plexus was based on the determination of a “capillary flow peak” signal derived from the B-scans. However, except for the DCP which is monoplanar, the SVP and the ICP are a 3D meshwork, so for instance, the outermost capillaries of the SVP are closer to the innermost capillaries of the ICP than presented in our analysis. Nevertheless, by identifying the highest peak of density and the distance between the capillary peaks, this method gave a good idea of the vascularisation of the GCL and INL. Lastly, we did not explore specifically the RPCP, which was therefore included in the calculation of the CD of the SVP, as we especially aimed to study the retinal capillary plexus distribution towards the retinal periphery.

In conclusion, using a wide montage of high resolution small-field angiograms obtained with SS-OCTA, we provided a detailed view of the retinal capillary network up to the far periphery in healthy eyes. We showed that the SVP and the DCP were both present up to the 11 mm of eccentricity analysed, while the ICP progressively vanished at about 8–9 mm from the foveal centre in all retinal sectors (with the exception of the nasal sector where it remained unchanged up to 10 mm). The density of the retinal capillary plexuses changed with changes in the retinal cellular structure. In the far periphery, the IPD increased just beyond the boundary of the ICP, an area where the non-perfusion usually starts in diabetic retinopathy^31–33^. Further studies are needed to build a reference base for studying the peripheral circulation in retinal vascular diseases.

## Supplementary information

Supplementary figures 1 and 2.
