# Supplementary material for: Retinal Capillary Plexus Pattern and Density from Fovea to Periphery Measured in Healthy Eyes with Swept-Source Optical Coherence Tomography Angiography
Source: Sci Rep. 2020 Jan 30;10:1474. doi: 10.1038/s41598-020-58359-y (PMC6992636; doi:10.1038/s41598-020-58359-y)
Supplement: Supplementary file 1 — Supplementary figures 1 and 2. [file 41598_2020_58359_MOESM1_ESM.pdf]

RETINAL CAPILLARY PLEXUS PATTERN AND DENSITY FROM FOVEA TO PERIPHERY MEASURED IN HEALTHY EYES WITH SWEPT-SOURCE OPTICAL COHERENCE TOMOGRAPHY ANGIOGRAPHY

Carlo Lavia, Pedro Mecê, Marco Nassisi, Sophie Bonnin, Jennifer Marie Louise, Aude Couturier, Ali Erginay, Ramin Tadayoni, Alain Gaudric.

SUPPLEMENTARY INFORMATION:

Supplementary figures 1 and 2

Supplementary figure 1

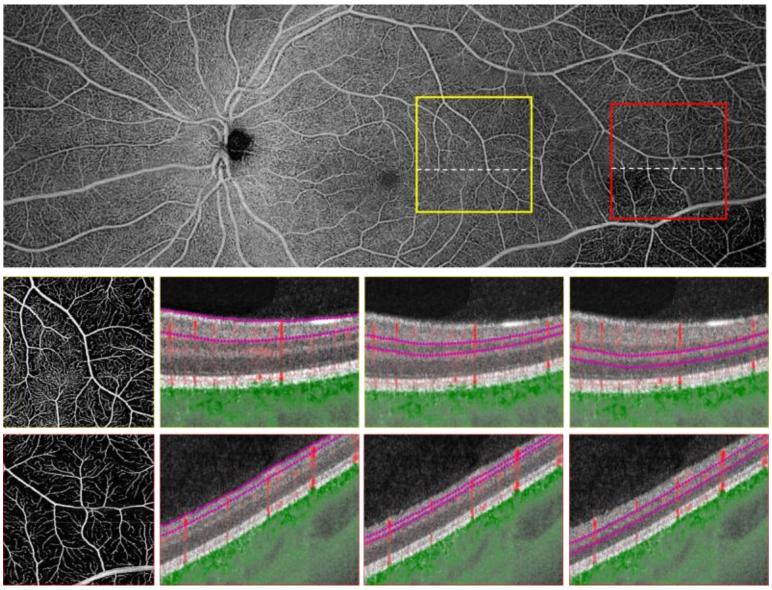

Segmentation lines on B-scans

First row: C-scan OCTA horizontal band obtained from a montage of five 12x12 mm OCTA scans, going nasally to the optic disc up to the temporal periphery. The yellow and red boxes with dotted lines are delineated to show the areas of equivalent 3x3 OCTA scans, centered at about 2 mm and 7 mm from the fovea (second and third rows).

Second and third row show on the left the 3x3 C-scan OCTA corresponding to the yellow and red boxes in the montage, respectively. The other boxes show the B-scans with flow overlay corresponding to the superficial vascular plexus, S(VP), the intermediate capillary plexus, (ICP) and the deep capillary plexus, (DCP). The purple lines define the inner and outer segmentation boundary used to isolate each plexus. The images have been magnified in the vertical axis to better show the relationships between the boundary and the flow signal.

Supplementary figure 2

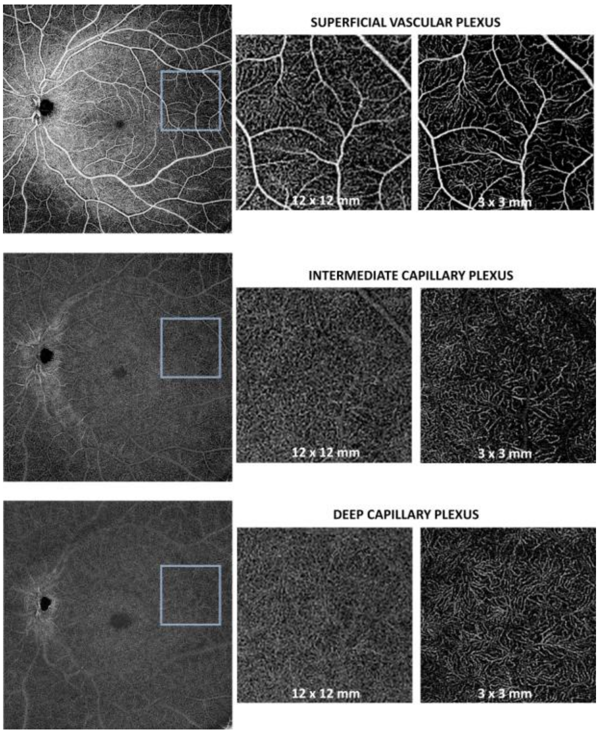

Comparison between wide and small field OCTA C-scans

First column: 12x12 mm C-scan OCTA centered on the fovea from the superficial vascular plexus (first row), intermediate capillary plexus (second row) and deep capillary plexus (third row). White boxes on the 12x12 mm C-scan OCTA indicate the location of the area of interest. Second column: magnification of the C-scan OCTA corresponding to the white boxes in the 12x12 mm scans. Third column: 3x3 mm C-scan OCTA corresponding to the same area. As compared to 12x12 mm scans, in the 3x3 mm scans the capillaries are better defined with clear capillary-free areas around precapillary arterioles in the superficial vascular plexus; the course of each capillary is easily followed in the intermediate and deep plexuses, that present different patterns, not distinguishable on the 12x12 mm scans.
